# Supplementary material for: Multi-omic identification of perineurial hyperplasia and lipid-associated nerve macrophages in human polyneuropathies
Source: Nat Commun. 2025 Aug 23;16:7872. doi: 10.1038/s41467-025-62964-8 (PMC12375038; doi:10.1038/s41467-025-62964-8)
Supplement: Supplementary file 1 — Supplementary Information [file 41467_2025_62964_MOESM1_ESM.pdf]

Supplementary Information

Multi-omic identification of perineurial hyperplasia and lipid-associated nerve macrophages in human polyneuropathies

Heming et al.

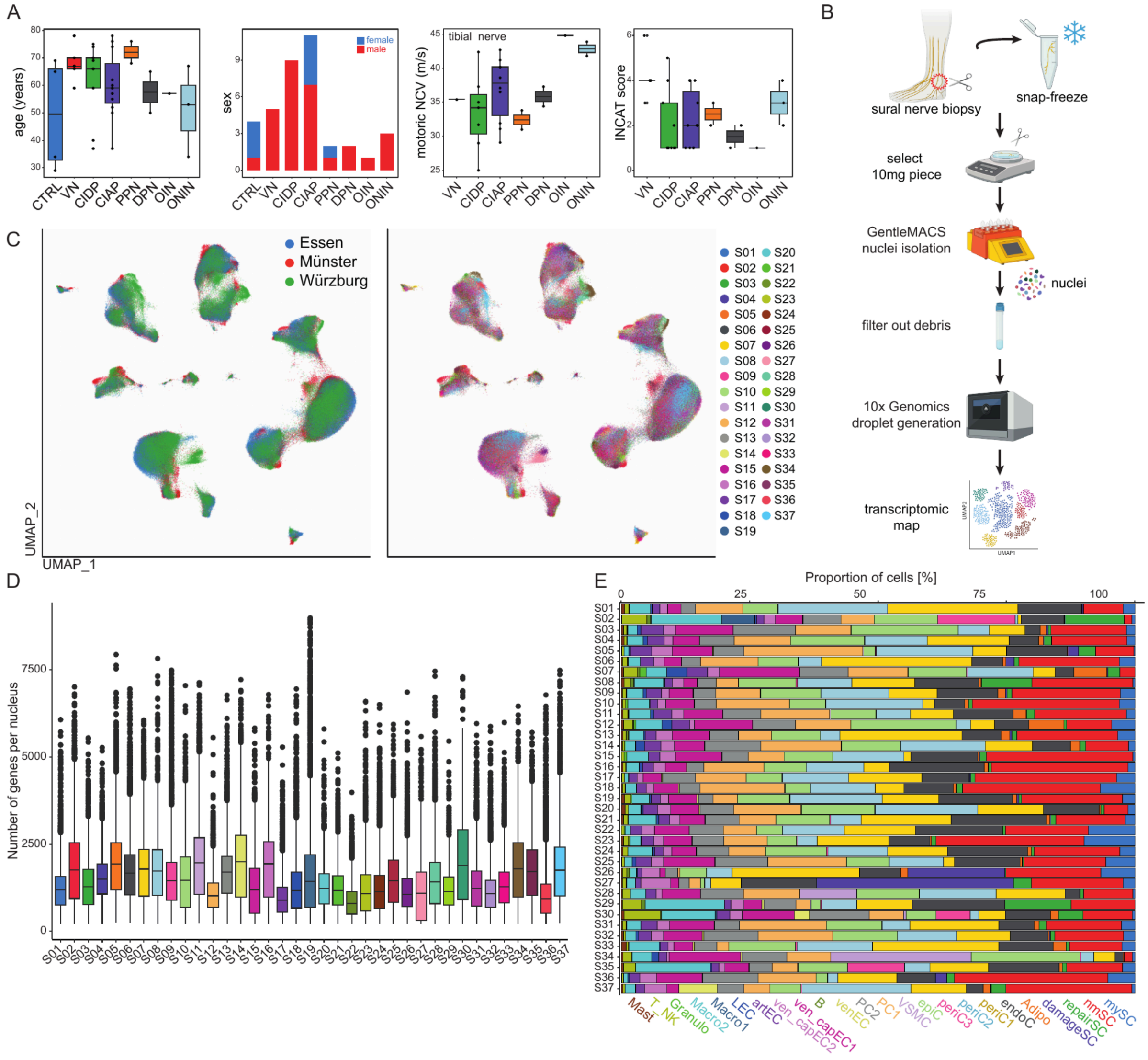

Supplementary Figure 1: Descriptive statistic of the snRNa-seq cohort and experimental quality metrics

(A) Average descriptive statistics of all patients (n = 37): average age in years, gender, results of the motoric test of nerve conduction velocity (NCV) in meters per second, and INCAT disability score are shown. Box plots show the median, interquartile range (IQR), and whiskers extending to 1.5 × IQR. Dots represent individual measurements. **(B)** Schematic illustrating the technical study design and the simplified nuclei purification protocol (methods). **(C)** UMAP of main clusters (Fig. 1B) split by center (left) or patients (right). **(D)** Number of genes per nucleus for each individual sample (n = 365,708 in total). **(E)** Proportion of cells split by samples and colored by cluster.

Icons were created in BioRender. Meyer zu Hörste, G. (2025) https://BioRender.com/a158762

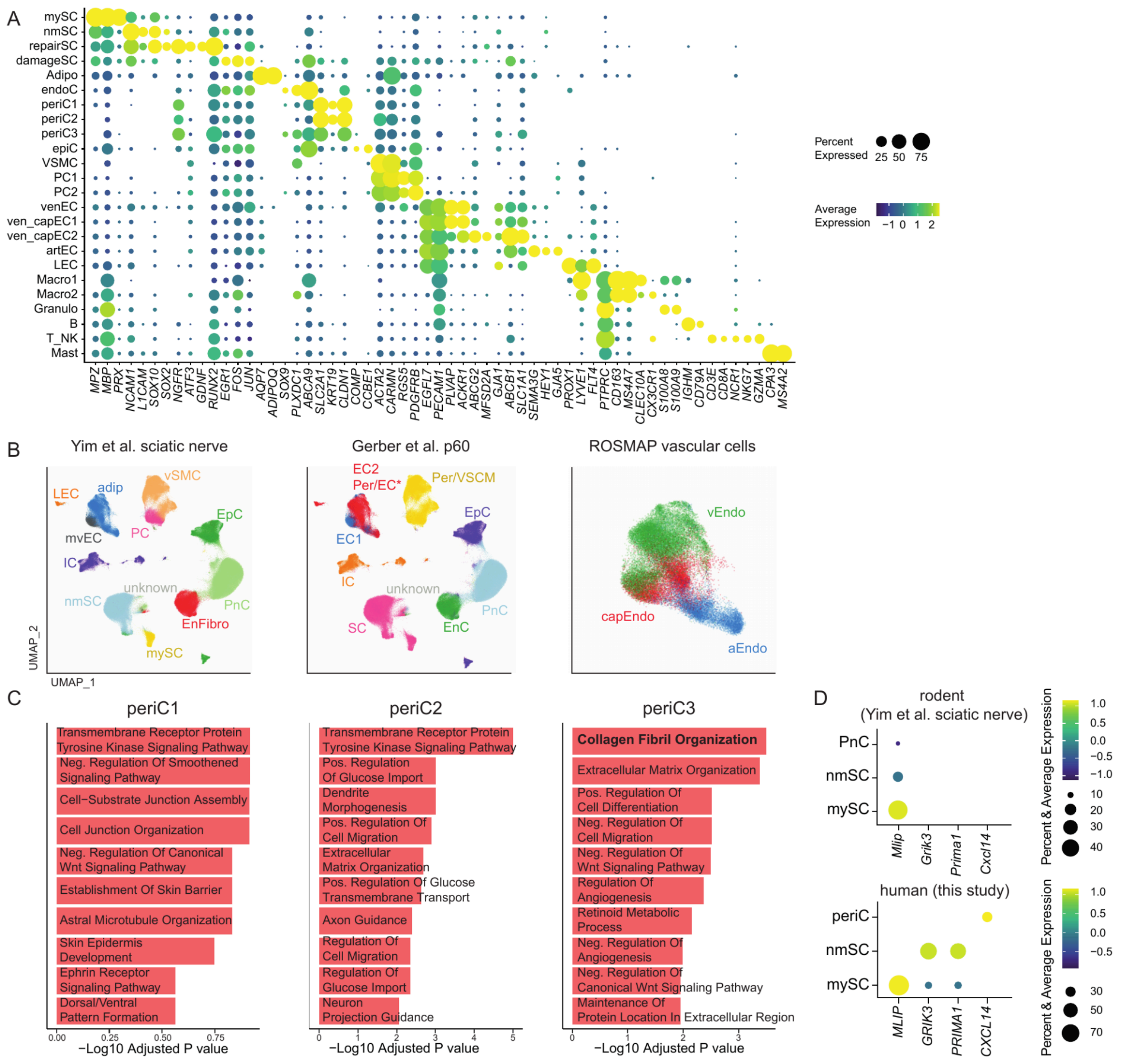

Supplementary Figure 2: Annotating nerve-associated cells in an unsupervised manner **(A)** Marker genes of main cell clusters (Fig. 1B). **(B)** UMAP of main clusters (Fig. 1B) with automatic annotation based on published rodent data (Yim et al., Gerber et al.). The rightmost plot shows vascular endothelial clusters only, automatically annotated with published human data (Mathy et al.). **(C)** Gene ontology term enrichment analysis of marker genes ($\log_2$ fold change > 2, adjusted p value < 0.001) expressed by the periC clusters in a one vs. all main cluster comparison. **(D)** Expression of novel cell marker genes in a published rodent dataset (Yim et al.) and this human study.

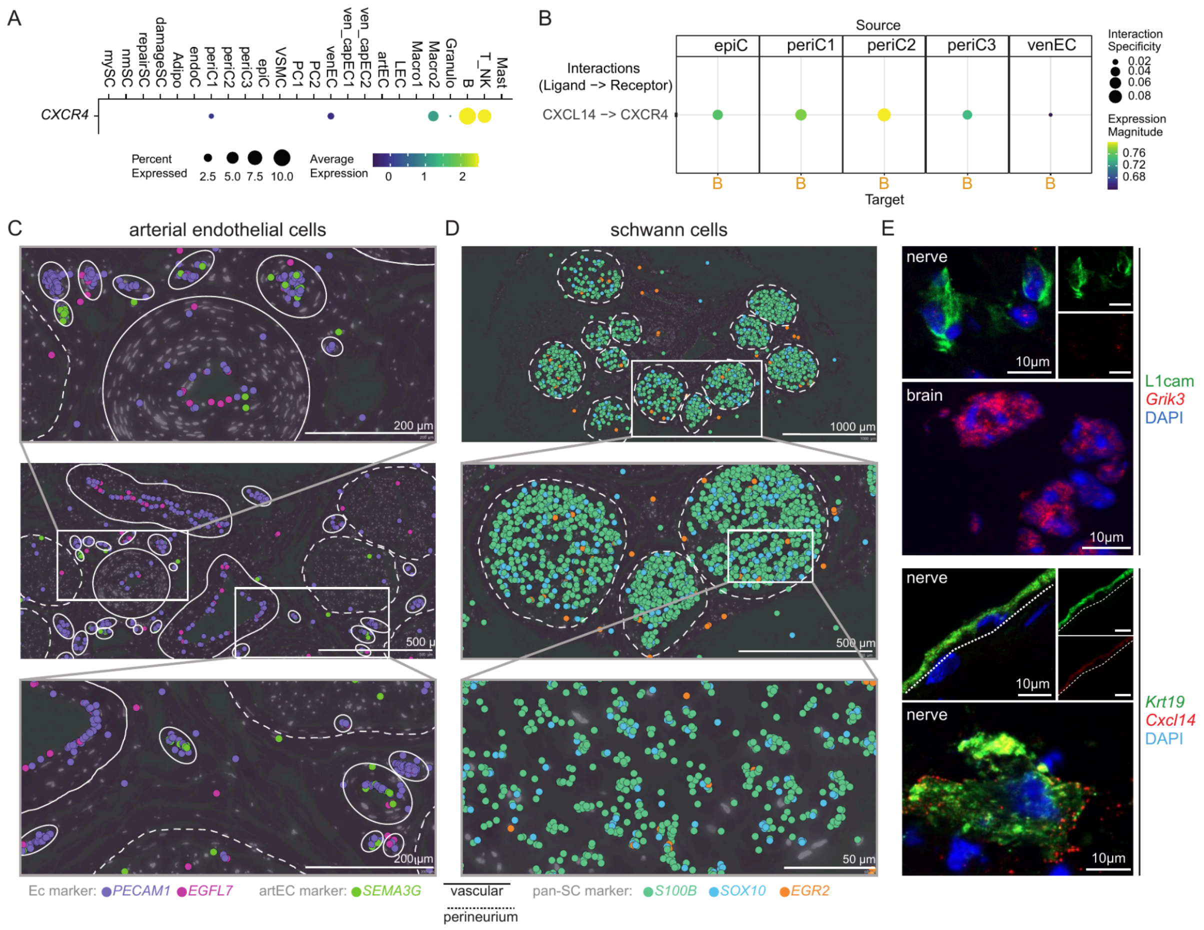

Supplementary Figure 3: Spatially characterizing peripheral nerves and confirming novel transcripts

(A) *CXCL14* expression in the depicted snRNA-seq cluster. **(B)** Ligand-receptor interactions involving *CXCL14* were computationally inferred from snRNA-seq gene expression and cluster annotation. **(C)-(D)** Spatial-seq was performed on sural nerve samples from a total of eight patients. Representative spatial transcriptomics images of (C) endothelial cell (Ec) markers and (D) Schwann cell (SC) markers in the sural nerve from a CTRL patient (S24). Each dot represents the expression of one transcript, a dotted line marks the perineurium, and a solid line surrounds the vasculature. **(E)** Combination of RNA *in situ* hybridization and immunofluorescence staining was performed in two sections in at least three independent experiments per marker combination. Representative images show expression of *Grik3* in murine non-myelating Schwann cells (L1cam-positive cells) and in brain cells as controls (upper panel) and the expression of *Cxcl14* in murine perineurial cells (*Krt19*-positive cells; lower panel). Nuclei were stained with DAPI.

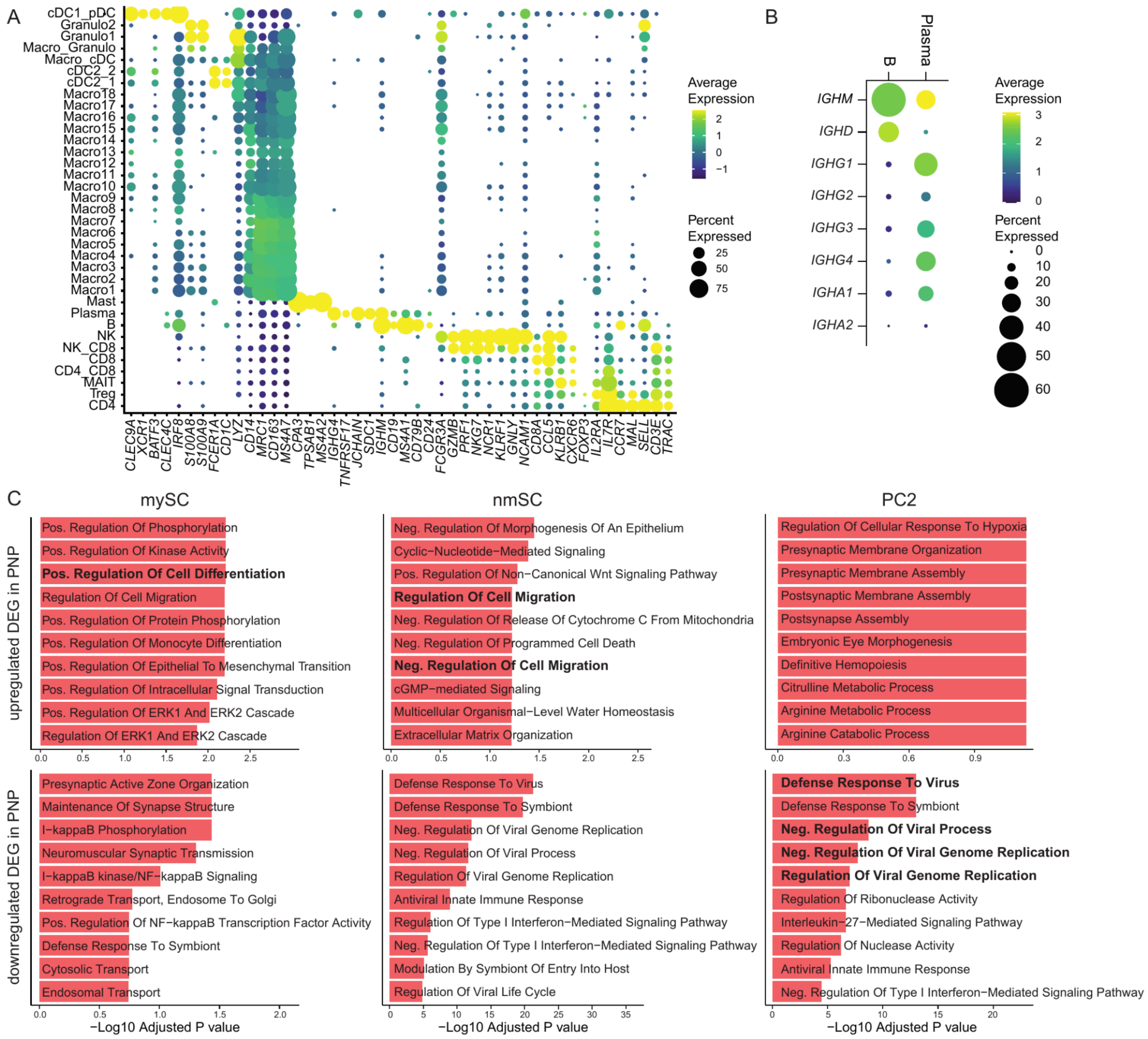

Supplementary Figure 4: Defining endoneurial immune cells

(A) Marker genes of immune cell clusters (Fig. 2A). **(B)** Expression of immunoglobulin heavy (*IGH*) chain genes in the B cell (B) and plasma cell (plasma) cluster of the IC subclusters. **(C)** Gene ontology term enrichment analysis of differentially expressed (DE) genes upregulated (upper panels; $\log_2$ fold change > 2, adjusted p value < 0.1) or downregulated (lower panels; $\log_2$ fold change < 2, adjusted p value < 0.1) in PNP compared to CTRL in myelinating Schwann Cells (mySC) (left), non-myelinating SC (nmSC) (middle), and pericytes 2 (PC2) (right) clusters. Statistical significance was assessed using enrichR (one-sided Fisher's exact test with p-values adjusted for multiple comparisons using the Benjamini-Hochberg method).

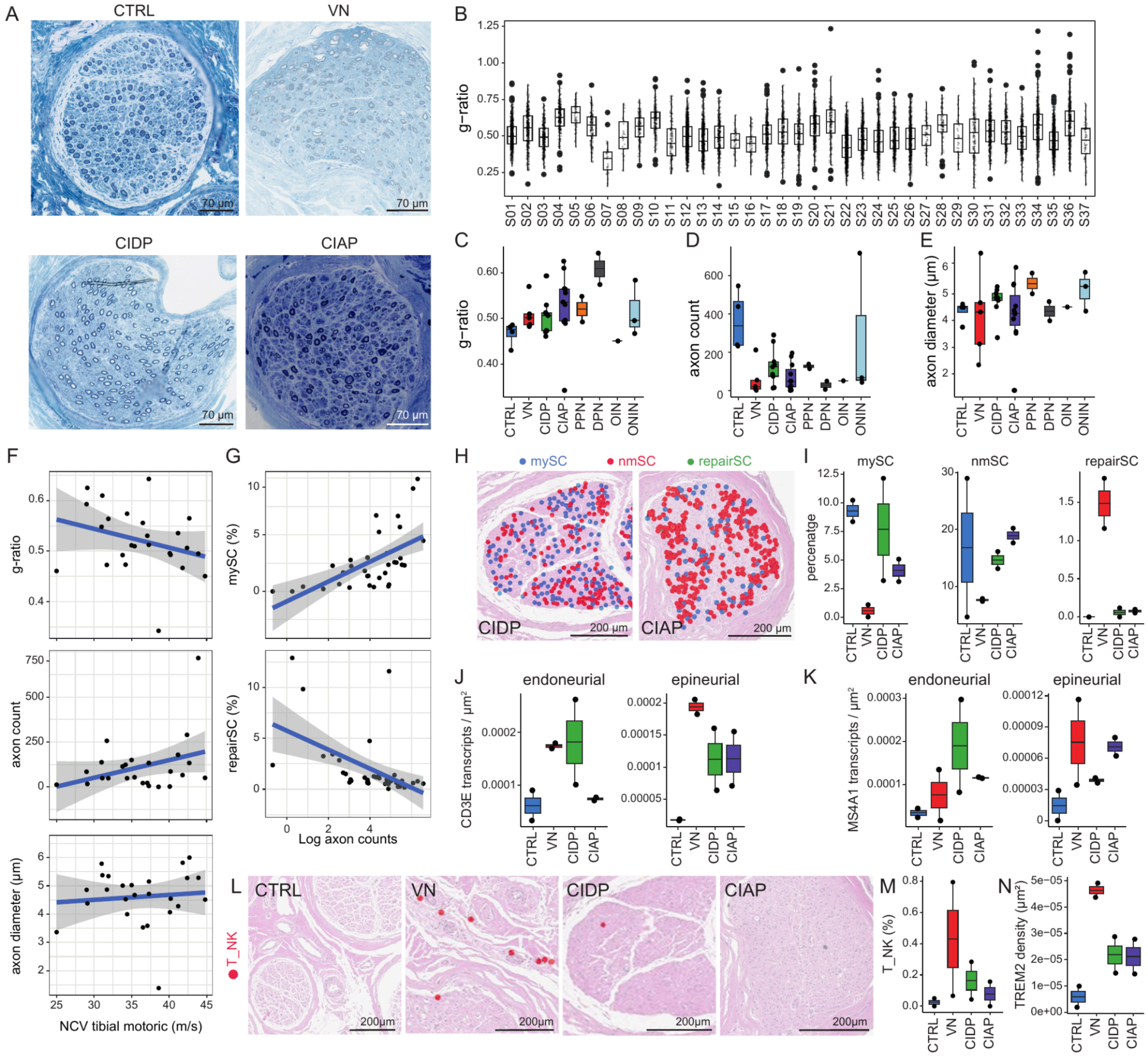

Supplementary Figure 5: Histological characterization of sural nerve biopsies

(A) Representative toluidine blue staining of the perineurium of CTRL (S24) VN (S30), CIDP (S01), and CIAP (S14) patients. Toluidine staining was performed in all 37 patients. **(B)** G-ratios (n = 11,076) quantified per sample (n = 37), with each dot representing an individual measurement. Box plots show the median, interquartile range (IQR), and whiskers extending to 1.5 × IQR. Dots represent individual measurements. **(C)-(E)** Histological measures (g-ratio, count of intactly myelinated axons, and diameter of axons) per disease group. Each dot represents the mean of the corresponding histological measure per patient (n = 37). **(F)** Correlation between g-ratio, acon count, and axon diameter of intactly myelinated axons with the motoric nerve conduction velocity (NCV) of the tibial nerve. Each dot represents one patient (n = 37). (**G**) Correlation between percentage of myelinating Schwann cells (mySC) and repair Schwann cells (repairSC) with the count of intactly myelinated axons. Each dot represents one patient (n = 37). **(H)** Spatial-seq was performed on sural nerve samples from a total of eight patients. Representative sections of H&E stainings of sural

nerves overlaid with spatial-seq showing predicted Schwann cell clusters in CIDP (S01) and CIAP (S14) patients. (**I**) Quantification of predicted mySC, nmSC, and repairSC clusters in spatial-seq in the four disease groups (n = 2 per group). **(J)-(K)** The density of (J) *CD3E* and (K) *MS4A1* transcripts in the endoneurial (left plot) and epineurial (right plot) per disease group (n = 2 per disease group). **(L)** Spatial-seq was performed on sural nerve samples from a total of eight patients (two per disease). Representative sections of H&E stainings of sural nerves overlaid with spatial-seq showing predicted T_NK cells in CTRL (S24), VN (S30), CIDP (S01), and CIAP (S14) patients. (**M**) Quantification of predicted T_NK cells in spatial-seq per disease group (n = 2 per group). **(N)** The density of *TREM2* transcripts in the entire nerve per disease group (n = 2 per disease group).
