# Supplementary material for: Multi-omic identification of perineurial hyperplasia and lipid-associated nerve macrophages in human polyneuropathies
Source: Nat Commun. 2025 Aug 23;16:7872. doi: 10.1038/s41467-025-62964-8 (PMC12375038; doi:10.1038/s41467-025-62964-8)
Supplement: Supplementary file 2 — Description of Additional Supplementary Files [file 41467_2025_62964_MOESM2_ESM.pdf]

Description of Additional Supplementary Files

File Name: Supplementary Data 1
Description: Patient information sequencing

File Name: Supplementary Data 2
Description: Technical information about single-nucleus RNA sequencing

File Name: Supplementary Data 3
Description: Top markers of this dataset of the main clusters

File Name: Supplementary Data 4
Description: Top markers of Yim et al. (sciatic nerve)

File Name: Supplementary Data 5
Description: Top markers of Gerber et al. (P60)

File Name: Supplementary Data 6
Description: Top markers of Wolbert et al. (mouse)

File Name: Supplementary Data 7
Description: Custom Xenium gene panel

File Name: Supplementary Data 8
Description: Top markers of this dataset of the immune cell clusters

File Name: Supplementary Data 9
Description: Differentially expressed genes in polyneuropathy vs. control patients per cluster

File Name: Supplementary Data 10
Description: Patient information EMA staining
