# Supplementary material for: Multi-omic identification of perineurial hyperplasia and lipid-associated nerve macrophages in human polyneuropathies
Source: Nat Commun. 2025 Aug 23;16:7872. doi: 10.1038/s41467-025-62964-8 (PMC12375038; doi:10.1038/s41467-025-62964-8)
Supplement: Supplementary file 13 — Reporting Summary [file 41467_2025_62964_MOESM13_ESM.pdf]

nature portfolio

Corresponding author(s): Gerd Meyer zu Hörste

Last updated by author(s): Jun 28, 2025

Reporting Summary

Nature Portfolio wishes to improve the reproducibility of the work that we publish. This form provides structure for consistency and transparency in reporting. For further information on Nature Portfolio policies, see our Editorial Policies and the Editorial Policy Checklist.

Statistics

For all statistical analyses, confirm that the following items are present in the figure legend, table legend, main text, or Methods section.

n/a	Confirmed	
☐	☒	The exact sample size (*n*) for each experimental group/condition, given as a discrete number and unit of measurement
☐	☒	A statement on whether measurements were taken from distinct samples or whether the same sample was measured repeatedly
☐	☒	The statistical test(s) used AND whether they are one- or two-sided<br>*Only common tests should be described solely by name; describe more complex techniques in the Methods section.*
☐	☒	A description of all covariates tested
☐	☒	A description of any assumptions or corrections, such as tests of normality and adjustment for multiple comparisons
☐	☒	A full description of the statistical parameters including central tendency (e.g. means) or other basic estimates (e.g. regression coefficient) AND variation (e.g. standard deviation) or associated estimates of uncertainty (e.g. confidence intervals)
☐	☒	For null hypothesis testing, the test statistic (e.g. *F*, *t*, *r*) with confidence intervals, effect sizes, degrees of freedom and *P* value noted<br>*Give P values as exact values whenever suitable.*
☒	☐	For Bayesian analysis, information on the choice of priors and Markov chain Monte Carlo settings
☒	☐	For hierarchical and complex designs, identification of the appropriate level for tests and full reporting of outcomes
☐	☒	Estimates of effect sizes (e.g. Cohen's *d*, Pearson's *r*), indicating how they were calculated

Our web collection on statistics for biologists contains articles on many of the points above.

Software and code

Policy information about availability of computer code

Data collection	Single nuclei RNA sequencing data were performed on Illumina Nextseq2000 and Novaseq 6000.<br>Spatial transcriptomics (Xenium) were performed on a Xenium Analyzer platform.
Data analysis	The code is publicly available at https://github.com/mihem/pns_atlas) and has been archived on Zenodo (https://doi.org/10.5281/zenodo.15750104). A Docker image is available to restore the coding environment. Figures are reproduced in a markdown document that automatically downloads all relevant source data from Zenodo available at https://mihem.github.io/pns_atlas/.<br><br>Software used: cellranger v7.0.1, CellBender v0.3.0 ImageJ v1.36 Xenium Explorer v1.3.0 TissUUmaps v3.1<br>R packages: Seurat v5.0.1 scDblFinder v1.16.0 scVI v1.0.4 enrichR 3.2 Libra v1.0.0 edgeR 4.0.7 Enhanced Volcano v1.20.0 miloDE v0.0.0.9 speckle v1.2.0 rcna v0.0.99 LIANA v0.1.14 homologene v.1.4.68 lme4 v1.1-35 cerebroAppLite v1.5.2

For manuscripts utilizing custom algorithms or software that are central to the research but not yet described in published literature, software must be made available to editors and reviewers. We strongly encourage code deposition in a community repository (e.g. GitHub). See the Nature Portfolio guidelines for submitting code & software for further information.

Data

Policy information about availability of data

All manuscripts must include a data availability statement. This statement should provide the following information, where applicable:

- Accession codes, unique identifiers, or web links for publicly available datasets
- A description of any restrictions on data availability
- For clinical datasets or third party data, please ensure that the statement adheres to our policy

The raw and processed single-cell sequencing data with sample and cluster annotations and spatial data generated in this study have been deposited in the GEO database under accession code GSE285983 (https://www.ncbi.nlm.nih.gov/geo/query/acc.cgi?acc=GSE285983) and GSE 285984 (https://www.ncbi.nlm.nih.gov/geo/query/acc.cgi?acc=GSE285984). An interactive version of the snRNA-seq data, created with cerebroAppLite v1.5.2 and an interactive version of the Xenium data, created with TissUUmaps v3.1, are available at https://pns-atlas.mzhlab.com. Source data are available on Zenodo (DOI:10.528, https://zenodo.org/records/15108216).

Research involving human participants, their data, or biological material

Policy information about studies with human participants or human data. See also policy information about sex, gender (identity/presentation), and sexual orientation and race, ethnicity and racism.

Reporting on sex and gender	The samples included in the study were collected from both male and female. This is shown in Supplementary Table 1. Sex was not included in the study design. No information on gender was collected.
Reporting on race, ethnicity, or other socially relevant groupings	We did not use the constructs of race and/or ethnicity in this study.
Population characteristics	Population characteristics are described in Supplementary Table 1 and visualized in Supplementary Fig. 1.
Recruitment	We collected sural nerves from 33 PNP patients from three centers: University Hospital of Münster (10 patients), Essen (11 patients), and Würzburg (12 patients). Additionally, we obtained sural nerves from four control patients in Essen with traumatic nerve injuries who received a sural nerve autograft as interposition but were unaffected by polyneuropathy (Supplementary Tab. 1).
Ethics oversight	All experiments were carried out in accordance with the Declaration of Helsinki and were approved by the local ethical committees in Münster (2018-719-f-S), Essen (21-10376-BO), and Würzburg (238/17; 15/19). All patients gave written informed consent to sample collection.

Note that full information on the approval of the study protocol must also be provided in the manuscript.

Field-specific reporting

Please select the one below that is the best fit for your research. If you are not sure, read the appropriate sections before making your selection.

☒ Life sciences ☐ Behavioural & social sciences ☐ Ecological, evolutionary & environmental sciences

For a reference copy of the document with all sections, see nature.com/documents/nr-reporting-summary-flat.pdf

Life sciences study design

All studies must disclose on these points even when the disclosure is negative.

Sample size	No sample size calculations were performed in advance. The samples size was determined based on previous scRNAseq studies and the limited availability of sural biopsies.<br><br>Our patient cohort is thus biased towards rare PNP etiologies and, for example, includes only two diabetic PNP, the most common cause of PNPs. We mitigate this by focusing on groups with at least four patients and analytically correcting for biases when possible.
Data exclusions	No samples were excluded. scRNAseq data were filtered based on mitochondrial percentages, molecule counts and doublet probability. Please refer to the methods section of the manuscript for details.
Replication	We have created a markdown document containing the code to reproduce the figures. https://mihem.github.io/pns_atlas/<br>The coding environment (i.e., the correct version of all packages and system dependencies) can be reliably restored using a Docker container.<br>All relevant source data are automatically downloaded from Zenodo (https://zenodo.org/records/15108216)<br>Analysis code is publically available via a GitHub repository (https://github.com/mihem/pns_atlas) archived on Zenodo https://doi.org/10.5281/zenodo.15750104)
Randomization	There was no randomization of samples.

Blinding	The physicians who recruited the patients were not blinded towards the diagnosis. Subsequent data analysis was performed in a pseudonymized and blinded fashion.

Reporting for specific materials, systems and methods

We require information from authors about some types of materials, experimental systems and methods used in many studies. Here, indicate whether each material, system or method listed is relevant to your study. If you are not sure if a list item applies to your research, read the appropriate section before selecting a response.

Materials & experimental systems

n/a	Involved in the study	
☐	☒	Antibodies
☒	☐	Eukaryotic cell lines
☒	☐	Palaeontology and archaeology
☐	☒	Animals and other organisms
☒	☐	Clinical data
☒	☐	Dual use research of concern
☒	☐	Plants

Methods

n/a	Involved in the study	
☒	☐	ChIP-seq
☒	☐	Flow cytometry
☒	☐	MRI-based neuroimaging

Antibodies

Antibodies used	L1cam (1:100, Abcam, ab24345), AF488 (1:100, Life, A-11001), Osteopontin (1:100, Abcam, ab63856), FABP5 (1:100, R&D Systems, AF1476), Perilipin-2 (1:100, Novusbio, NBP2-48532), CD36 (1:100, Abcam, ab124515), CXCL14 (1:100, Abcam, ab264467) EMA (Dako, clone E29, undiluted)
Validation	Antibodies used are commercially available and were validated by the manufacturer.

Animals and other research organisms

Policy information about studies involving animals; ARRIVE guidelines recommended for reporting animal research, and Sex and Gender in Research

Laboratory animals	C57BL/6J (WT) mice were purchased from Charles River.
Wild animals	The study did not involve wild animals.
Reporting on sex	Female mice at the age of 10-15 weeks were used.
Field-collected samples	The study did not involve field-collected samples.
Ethics oversight	All animal experiments were approved by the responsible state authority (Landesamt für Natur, Umwelt und Verbraucherschutz Nordrhein-Westfalen) and the ethics committees of the University Münster and were performed in accordance with local regulations.

Note that full information on the approval of the study protocol must also be provided in the manuscript.

Plants

Seed stocks	/
Novel plant genotypes	/
Authentication	/
